# Key genes and pathogenesis related to type 2 diabetes and cardiogenic stroke

**DOI:** 10.1097/MD.0000000000044242

**Published:** 2025-09-05

**Authors:** Zhiyuan Deng, Manjia Wang, Hongli Song, Min Liu

**Affiliations:** a Department of Endocrinology, Gaozhou Hospital of Traditional Chinese Medicine, Gaozhou, China; b Department of Gynecology, Gaozhou Hospital of Traditional Chinese Medicine, Gaozhou, China; c Department of Endocrinology, The First Affiliated Hospital of Guangzhou University of Traditional Chinese Medicine, Guangzhou, China.

**Keywords:** bioinformatics analysis, cardiogenic stroke, hub gene, Mendelian randomization analysis, type 2 diabetes

## Abstract

Type 2 diabetes mellitus (T2DM) and cardiogenic stroke (CS) are harmful to human health. Previous studies have shown a correlation between T2DM and CS, but the causal relationships and pathogenic mechanisms between T2DM and CS remain unclear. We downloaded T2DM and CS datasets from a genome-wide Association Study and performed Mendelian randomization (MR) analysis using the TwoSampleMR package in R software. To obtain differentially expressed genes, The Limma package of R software was used to analyze the T2DM and CS datasets. Both datasets were acquired from the Gene Expression Omnibus. Gene Ontology and Kyoto Encyclopedia of Genes and Genomes were used to analyze co-driver genes for pathway enrichment. On the basis of protein–protein interaction network and Interaction Gene Retrieval Tool database, the hub genes were founded using Cytoscape software. The regulatory relationship between microRNAs (miRNAs) and hub genes was demonstrated using NetworkAnalyst. The Cytoscape plugin, CytoHubba tool, was employed to screen hub genes and evaluate common diagnostic markers for T2DM and CS. The MR analysis showed a correlation between T2DM and CS. T2DM increased the risk of CS (*P *= .003, inverse-variance weighted method). There was no statistical heterogeneity among single nucleotide polymorphisms strongly associated with T2DM (Cochran’s *Q* = 130.48, *P* = .139) and no horizontal pleiotropy among single nucleotide polymorphisms strongly associated with T2DM (*P *= .677, MR-Egger regression). We predicted that miR-34a-5p, miR-103-3p, miR-107, and miR-124-3p may be key miRNAs in the miRNA-gene network. This study suggested that T2DM increased the risk of CS, and T2DM and CS share common diagnostic biomarkers and pathogenic pathways.

## 1. Introduction

Stroke is the second leading cause of death and third leading cause of disability worldwide. With its rising incidence, disability, and mortality rates, stroke has caused significant harm to global health.^[1]^ Cardiogenic stroke (CS) accounts for 20% to 25% of all ischemic stroke cases.^[2]^ Compared with other stroke subtypes, CS is more severe, and prognosis is worse.^[3]^ Currently, research on the risk factors for CS mainly focuses on cardiac self-lesions and vascular lesions; however, there are many controversies. In 2020, Fill et al^[4]^ validated the causal relationship between the genetic susceptibility to atrial fibrillation and stroke in a Mendelian randomization (MR) study. Further research of stroke subtypes revealed that atrial fibrillation was mainly associated with CS (*P* < .0001) but not with other stroke subtypes. In addition, another MR study reached the same conclusion.^[5]^ Both studies provided causal evidence from a genetic perspective on the association of atrial fibrillation with CS. Bidirectional and multivariate MR evaluations^[6]^ were conducted to investigate the mutual influence of heart failure on the subtypes of ischemic stroke. The results showed that heart failure had a causal relationship with large atherosclerotic stroke (*P* < .0001) and CS (*P* = .044). However, the results were not robust due to interference from cardiovascular disease and atrial fibrillation.^[7]^ The MR data of Tian et al also supported the causal relationship between myocardial infarction and large atherosclerotic stroke (*P* < .0001), and ischemic stroke (*P* = .002) but not CS.^[5]^ Another study found that after excluding diabetes, hypertension, and cardiovascular disease, the correlation between coronary occlusion and stroke was no longer significant. Moreover, the risk factors for CS have not yet been determined.^[8]^

At the present time, many clinical studies have found that diabetes is related to the occurrence and aggravation of cardiovascular and cerebrovascular diseases, and that an increase in blood sugar variability often worsens the outcomes of cardiovascular diseases.^[9–11]^ However, the correlation and causal relationship between diabetes and CS have not yet been determined. A meta-analysis showed that diabetes doubled the risk of cardiovascular disease and was independent of other cardiovascular risk factors.^[12]^ However, this study did not further analyze the impact of different types of diabetes. Cai et al carried out a meta-analysis involving 10 million participants. The results showed that after an average follow-up of 9.8 years, prediabetes can significantly increase the cardiovascular risk (relative risk of all-cause death: 1.13, 95% confidence interval [CI]: 1.10–1.17; cardiovascular disease, RR: 1.16, 95% CI: 1.11–1.21; stroke, RR: 1.14, 95% CI: 1.08–1.20).^[13]^ Another study analyzed the risk factors for CS. The single factor analysis showed that the related factors of patients with cardiogenic disease complicated with stroke included gender, age, previous stroke history, history of transient ischemic attack, history of systemic embolism, history of coronary heart disease, heart dysfunction, hypertension, diabetes, vascular disease, mitral stenosis, artificial valves, heart failure, LVEF ≤ 30%, hyperthyroidism, and congestive heart failure. Multivariate logistic regression results found that the injury factors of people with cardiogenic disease complicated with stroke were age ≥75 years, previous stroke history, history of transient ischemic attack, history of systemic embolism, moderate and severe LV dysfunction, but not diabetes.^[14]^ These results indicate that diabetes is related to cardiovascular diseases and stroke; however, there is no direct evidence that type 2 diabetes mellitus (T2DM) has a causal relationship with CS.

CS and T2DM are 2 diseases that significantly affect human physical and mental health and often occur together. Therefore, investigating the mechanisms underlying T2DM and CS is considerable. This study aimed to discover the correlation between T2DM and CS based on MR analysis. On the basis of Gene Expression Omnibus (GEO) database, we filtrated candidate co-driver genes for T2DM and CS. Through Gene Ontology (GO) and Kyoto Encyclopedia of Genes and Genomes (KEGG), we explored mutual biological pathways. We also identified hub genes and evaluated their predictive values.

## 2. Methods

### 2.1. Gene chip data acquisition

With T2DM as the exposure factor and CS as the outcome, MR Analysis was used to investigate the causality between T2DM and CS. The T2DM and CS datasets were provided from IEU OPEN GWAS (https://gwas.mrcieu.ac.uk/) and all were Europeans. The T2DM dataset (ID: the sample size of ebi-a-GCST006867) was 6,55,666 cases with 50,30,727 single nucleotide polymorphisms (SNPs). The sample size of the CS dataset (ID: ukb-b-8714) was 4,61,880 cases, and the SNPs were 98,51,867. In this study, SNPs were used as instrumental variables. The specific screening process was as follows: in step 1 we set *P*-value to be <5 × 10^−8^ to screen SNPs that were strongly correlated with T2DM^[15]^; in the second step, we set the threshold of the linkage disequilibrium parameter to .001 and the genetic distance to 10,000 kb to ensure that the SNP was not subject to any confounding factors related to the outcome^[16]^; the third step was to further screen SNPs through the phenoscanner database to remove the influence of known confounding factors and ensure that the SNPs affected outcomes only through exposure factors^[17]^; in step 4 we eliminated palindromic SNPs, selected SNPs with *F*-statistics >10 [*F*-statistic = (β/SE)^2^], and eliminated weak instrumental variables as much as possible.

The application for 2-sample MR analysis was the TwoSampleMR package in R software. The inverse variance-weighted (IVW) method was used for the main MR analysis. The MR-Egger regression weighted median method, simple model, and weighted model were the secondary methods.^[18]^ To investigate the causality of T2DM with CS, a *P*-value <.05 was considered statistically significant. For evaluating the statistical heterogeneity among SNPs that were strongly correlated with T2DM, Cochran’s *Q* test was employed. There was no statistical heterogeneity among SNPs with *P* > .05, and a fixed-effects model was used to estimate causality. If *P *< .05, a random-effects model was used to estimate causality.^[19,20]^ The intercept term of the MR-Egger regression and Mendelian Randomization Pleiotropy RESidual Sum and Outlier (MR-PRESSO) were used to evaluate the horizontal pleiotropy of SNPs strongly related to T2DM, and *P* > .05 suggested no horizontal pleiotropy.^[21]^ The influence of a single SNP on MR results was identified by leave-one-out method.

Two microarray datasets (GSE95849 and GSE58294) for T2DM, CS were selected from GEO database based on GPL22449 and GPL570-55999 platforms. The GSE95849 dataset included gene expression profiles from 12 T2DM patients and 6 healthy individuals. The GSE58294 dataset contained 69 CS patients and 23 controls.

### 2.2. Acquisition of differentially expressed genes

The R software package “limma” was used to extract and analyze the differentially expressed genes (DEGs). The DEGs screening threshold was *P* < .05, |log_2_ FC|>1. We used Benjamini-Hochberg method to adjust the *P*-values for multiple testing. Packages “pheatmap” and “ggplot2” in R software were used to visualize the temperature gradients of the 2 datasets respectively and creat heat and volcano maps. R packages “venn” and “VennDiagram” were used to depict common DEGs between T2DM and CS. Based on the analysis platform (https://cloud.keyandaydayup.com/), we conducted principal component analysis.

### 2.3. Biological functions enrichment of DEGs

The above common DEGs were analyzed by GO functional enrichment analysis, including biological process, cell component, and molecular function. KEGG signaling pathway analysis was implemented by the R software package “clusterProfiler.” Gene set enrichment analysis (GSEA)was implemented by the GSEA software (version 4.4.0). Adjusted *P* value <.05 was set as the selection criterion for enriched pathways.

### 2.4. Identifying protein–protein interaction (PPI) hub genes

We constructed a protein–protein interaction (PPI) mesh utilizing the Interaction Gene Retrieval Tool (http://string-db.org/) to analyze the interactions between the hub genes. The minimum interaction score was set to 0.4. The PPI Netscape was visualized using Cytoscape software. Subsequently, we then used the Cytoscape plugin MCODE to screen for key protein expression molecules. Finally, we used the maximum clique centrality algorithm in the CytoHubba plugin to screen hub genes with high connectivity.

### 2.5. Screening for hub gene-related miRNAs

NetworkAnalyst (version 3.0, https://www.networkanalyst.ca/) was used to construct the microRNAs (miRNA)-gene interaction map of the hub genes. Selected hub genes and miRNAs were shown by Cytoscape software.

## 3. Results

### 3.1. MR Results of 2 samples

**Figure 1.**
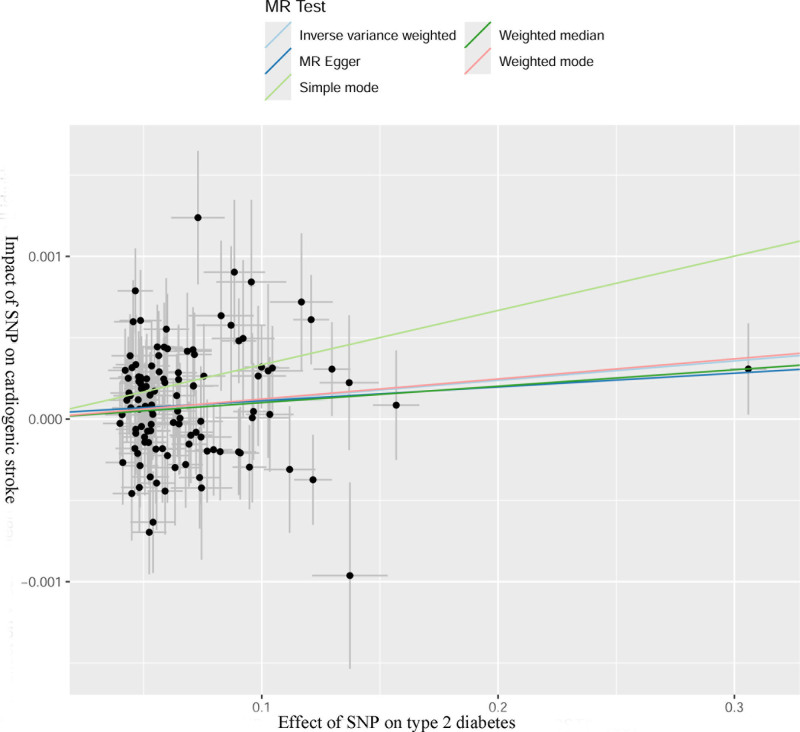
Relation between T2DM and CS shown in scatter plot analysis. CS = cardiogenic stroke, MR = Mendelian randomization, SNP = single nucleotide polymorphism, T2DM = type 2 diabetes mellitus.

**Figure 2.**
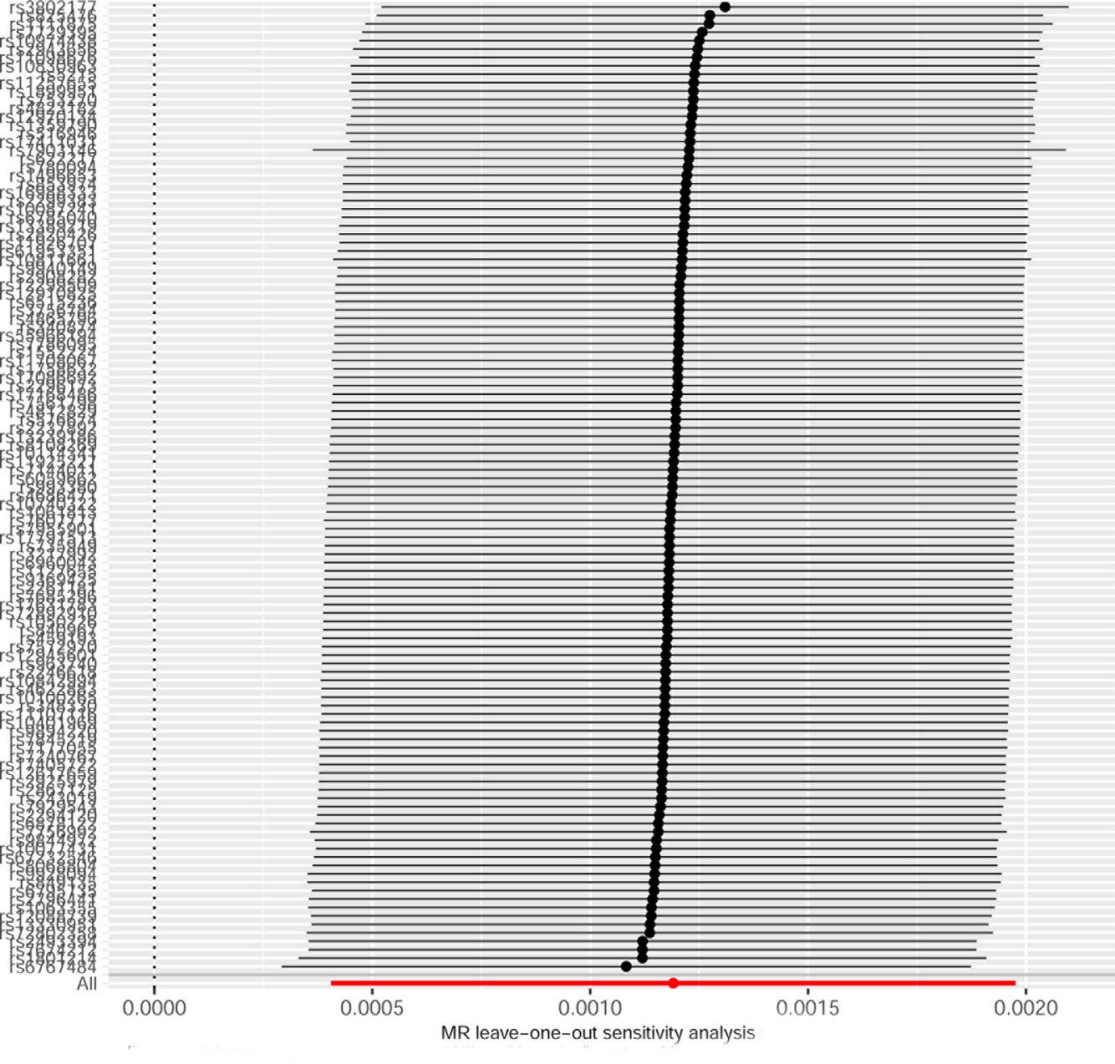
Results of a leave-one-out analysis showing the causal link between T2DM and CS. CS = cardiogenic stroke, MR = Mendelian randomization, T2DM = type 2 diabetes mellitus.

**Figure 3.**
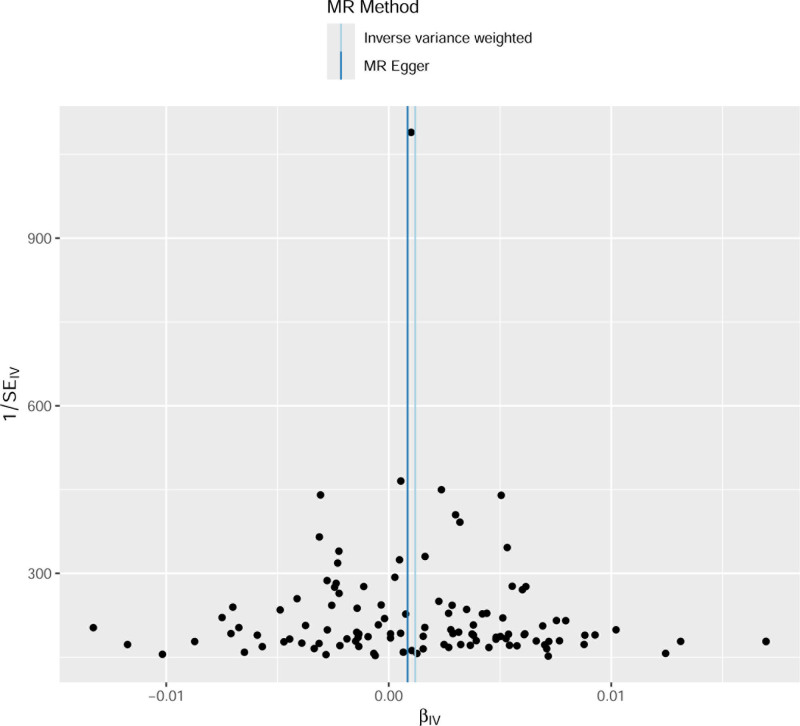
Funnel plot of SNPs strongly associated with T2DM. MR = Mendelian randomization, SNPs = single nucleotide polymorphisms, T2DM = type 2 diabetes mellitus.

In total, 115 SNPs strongly associated with T2DM were screened in this study. *F*-statistics of 115 SNPs were all >10, indicating that the impact of weak instrumental bias was minimal, and no SNPs containing confounding factors were found. This was to ensure that the selected SNPs were highly correlated with T2DM. T2DM enhanced the risk of CS (IVW analysis, odds ratio = 1.0012, 95% CI (1.0004–1.0020), *P *= .003). Additionally, the simple model analysis also showed similar results (*P* = .048). The results of the MR-Egger regression, weighted median method, and weighted model were not statistically significant (*P* > .05), as shown in Table 1 and Figure 1. Normally, we relied on the results of IVW analysis. There was no statistical heterogeneity among the SNPs (Cochran’s *Q* = 130.48, *P* = .139). Intercept term analysis of the MR-Egger regression and MR-PRESSO showed that the SNPs that were strongly associated with T2DM did not have horizontal pleiotropy, and the *P*-values were .677 and.135, respectively. This was to ensure that the selected SNPs only affected the onset of CS through T2DM, meeting exclusivity requirement. The results of the leave-one-out method showed that the MR results were robust, excluding the excessive influence of a single SNP (Fig. 2). The funnel plot showed that the MR analysis results were less affected by potential factors (Fig. 3).

**Table 1:** MR analysis of T2DM/CS causal relationship.

MR analysis method	β	OR	95% CI	*P*-value
IVW	0.001191	1.001191	1.0004–1.0020	.002962
MR-Egger	0.000847	1.000848	0.9991–1.0026	.355966
Weighted median	0.00101	1.001011	0.9995–1.0025	.188564
Simple mode	0.003339	1.003344	1.0001–1.0066	.047826
Weighted mode	0.001233	1.001233	0.9997–1.0028	.128193

CI = confidence interval, CS = cardiogenic stroke, IVW = inverse variance-weighted, MR = Mendelian randomization, OR = odds ratio, T2DM = type 2 diabetes mellitus.

### 3.2. DEGs in T2DM and CS

**Figure 4.**
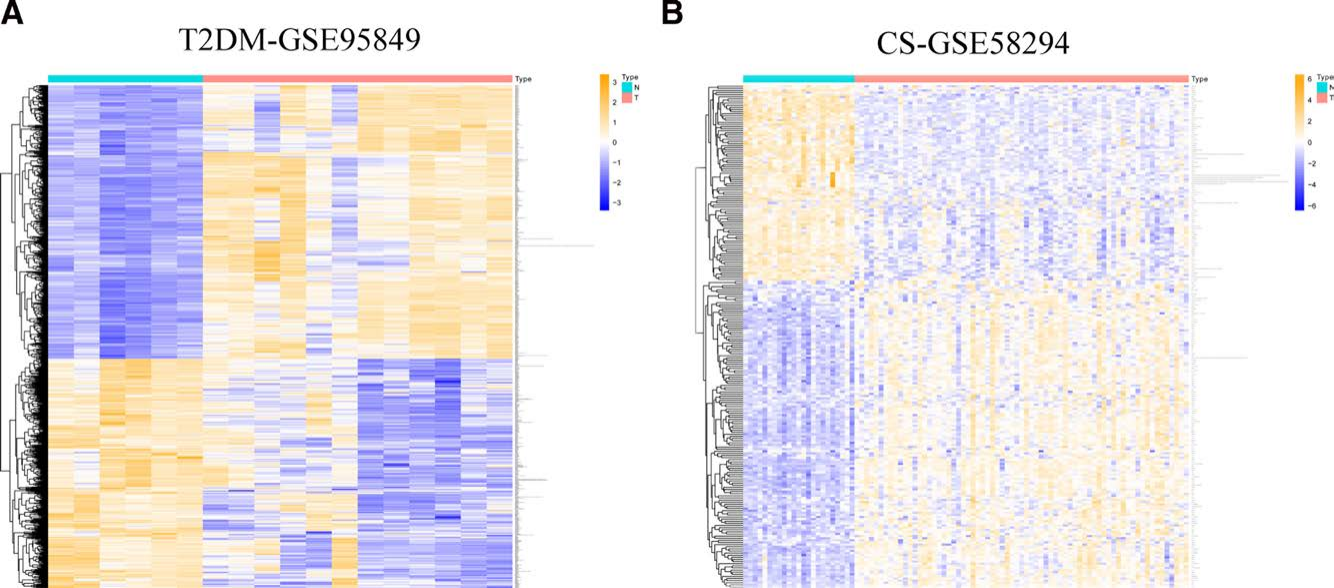
Heatmaps of differentially expressed genes (DEGs). Panel (A), heatmap of DEGs in GSE95849; panel (B), heatmap in GSE58294. Red indicates upregulation of DEGs, blue indicates downregulation of DEGs, and white indicates no significant change. CS = cardiogenic stroke, DEGs = differentially expressed genes, T2DM = type 2 diabetes mellitus.

**Figure 5.**
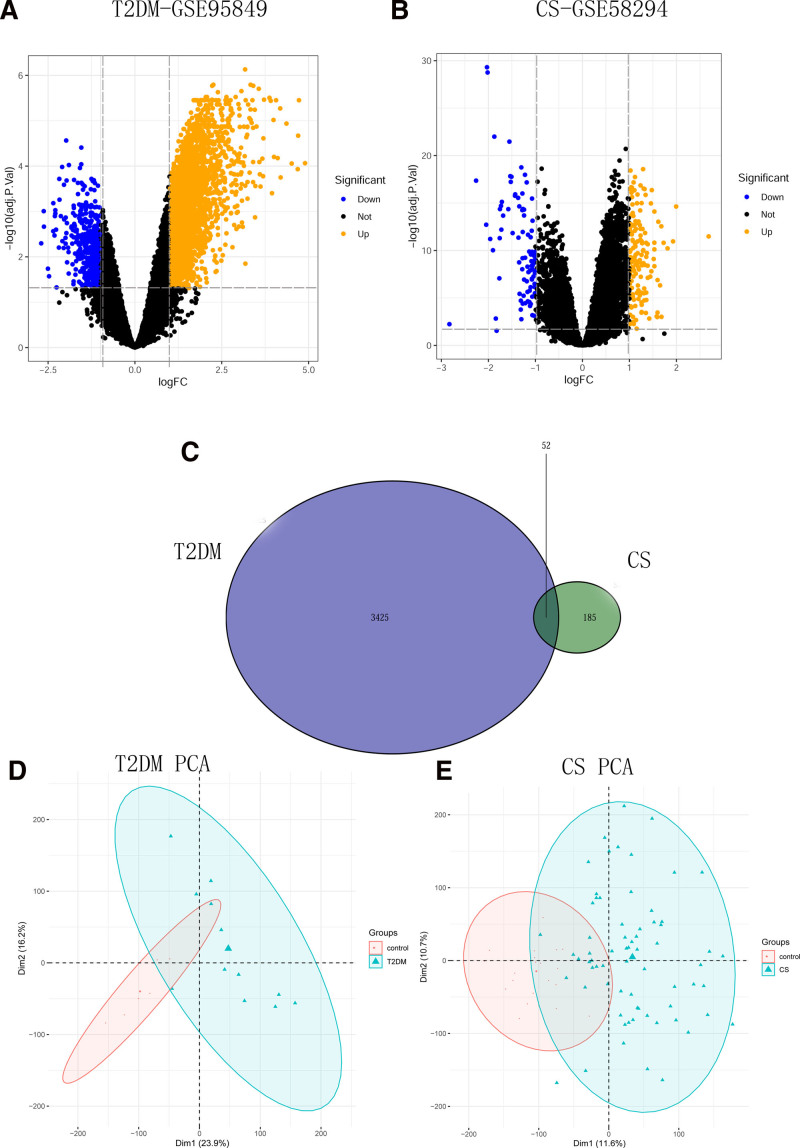
Analysis of differentially expressed genes (DEGs) in the datasets GSE95849 and GSE58294. (A, B) Figures illustrate the volcano plots displaying the DEGs in each dataset, while (C) figure shows a Venn diagram comparing the DEGs between the 2 datasets. The vertical dashed lines in the diagrams represent the log_2_ FC values of −1 and 1, respectively, whereas the horizontal line represents an adjusted *P*-value of .05. (D, E) Figures illustrate the PCA plots in each dataset. The horizontal axis represents the contribution rate of the first principal component, the vertical axis represents the contribution rate of the second principal component, each point represents a sample, and the circle range represents the degree of separation of the samples. CS = cardiogenic stroke, DEGs = differentially expressed genes, PCA = principal component analysis.

The GSE95849 dataset for T2DM and the GSE58294 dataset for CS were downloaded from the GEO database. After adjusting the threshold screening for *P* < .05, |log_2_ FC| > 1.0, 3077 genes (3021 upregulated and 456 downregulated) were identified in the GSE95849 dataset. There were altogether 237 genes selected in the GSE58294 dataset (145 upregulated and 92 downregulated genes). The DEGs were visualized using volcano and heat maps, as shown in Figures 4 and 5, respectively. Principal component analysis displayed the contribution rate of principal components and the degree of separation between samples, shown in Figure 5D, E. Additionally, a Venn diagram of the common DEGs between GSE95849 and GSE58294 was constructed, shown in Figure 5C. In total, 52 overlapping DEGs were identified.

### 3.3. Enriched pathways for overlapping DEGs

We selected the top 10 words that were significantly enriched in GO and KEGG with an adjusting *P*-value <.05.

**Figure 6.**
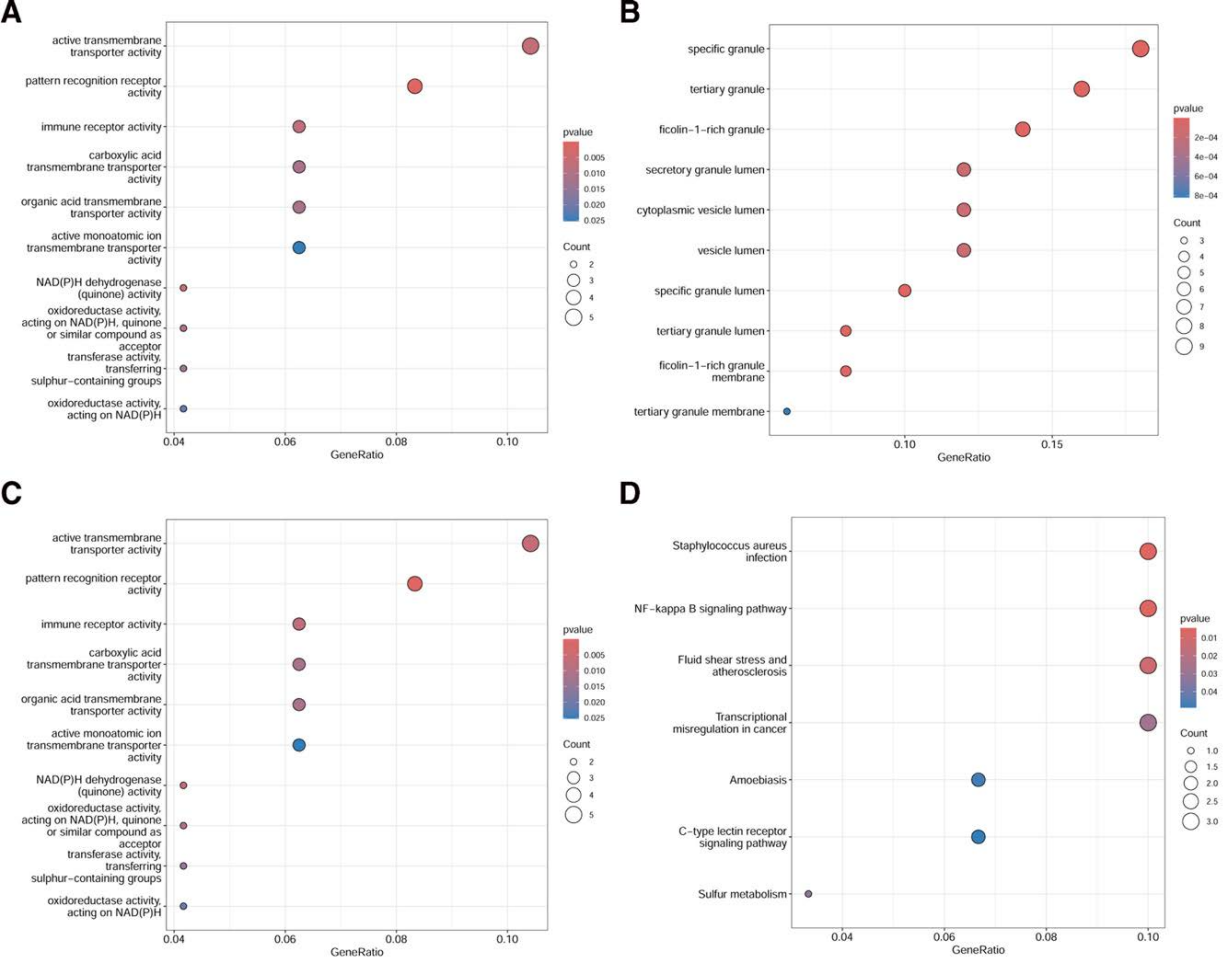
Results of the analysis conducted on overlapping DEGs, utilizing GO and KEGG enrichment. The results of the analysis are depicted for 4 distinct sections. (A) Figure showcases the enrichment findings related to GO biological processes. (B) Figure shows the results of the enrichment of GO cell components. (C) Demonstrates the functional enrichment outcomes for the GO molecules. (D) Figure illustrate the KEGG pathway enrichment results. A correction was made to ensure the validity of the results, with *P *< .05 signifying noteworthy changes in GO and KEGG. The *x*-axis of the figures denotes the proportion of genes related to each specific item, whereas the *y*-axis reflects the annotation items of GO-BP, GO-CC, GO-MF, and KEGG. Size and color of bubbles represent the number of genes associated with each item and the adjusted *P*-value, with a deeper red shade indicating a higher enrichment level. DEGs = differentially expressed genes, GO = Gene Ontology, KEGG = Kyoto Encyclopedia of Genes and Genomes Pathway Enrichment Analysis.

**Figure 7.**
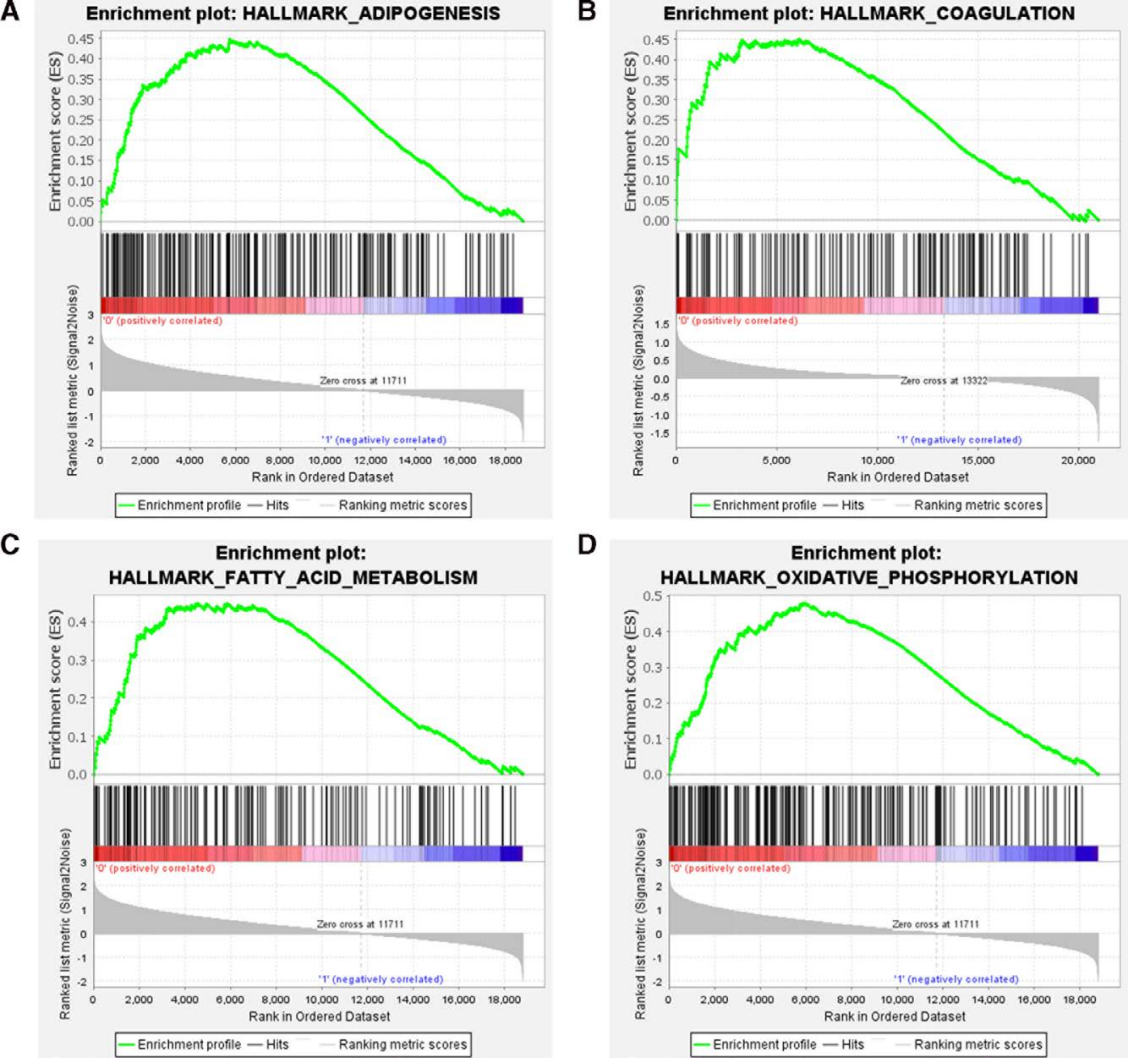
GSEA enrichment results. Panel (A), GSEA enrichment in adipogenesis. Panel (B) shows the GSEA enrichment in coagulation. Panel (C) shows the GSEA enrichment in fatty acid metabolism. Panel (D) shows the GSEA enrichment in oxidative phosphorylation. GSEA = gene set enrichment analysis.

The DEGs were enriched in biological processes, including the regulation of monoatomic ion transport, regulation of transmembrane transport, defense response to bacteria, regulation of monoatomic ion transmembrane transport, immune response-regulating cell surface receptor signaling pathway, positive regulation of response to biotic stimulus, defense response to fungus, response to fungus regulation of action potential, and modulation of process of another organism (Fig. 6A). In terms of cellular composition, the DEGs were mainly related to specific granule, tertiary granule, ficolin-1-rich granule, secretory granule lumen, cytoplasmic vesicle lumen, vesicle lumen, specific granule lumen, tertiary granule lumen, ficolin-1-rich granule membrane, and tertiary granule membrane (Fig. 6B). The molecular functional analysis showed that the DEGs were related to active transmembrane transporter activity, pattern recognition receptor activity, immune receptor activity, carboxylic acid transmembrane transporter activity, organic acid transmembrane transporter activity, active monoatomic ion transmembrane transporter, NAD(P)H dehydrogenase (quinone) activity, oxidoreductase activity acting on NAD(P)H, quinone or similar compounds as acceptors, transferase activity, transferring sulfur-containing groups, and oxidoreductase activity acting on NAD(P)H (Fig. 6C). The top 10 significant KEGG pathways in DEGs were enriched in *staphylococcus aureus* infection, NF-κB signaling pathway, fluid shear stress and atherosclerosis, transcriptional misregulation in cancer, amebiasis, C-type lectin receptor signaling pathway, and sulfur metabolism (Fig. 6D). The GSEA analysis results showed that the genes were mainly enriched in fatty acid metabolism, adipogenesis, oxidative phosphorylation, and coagulation (Fig. 7).

### 3.4. PPI network of hub genes

**Figure 8.**
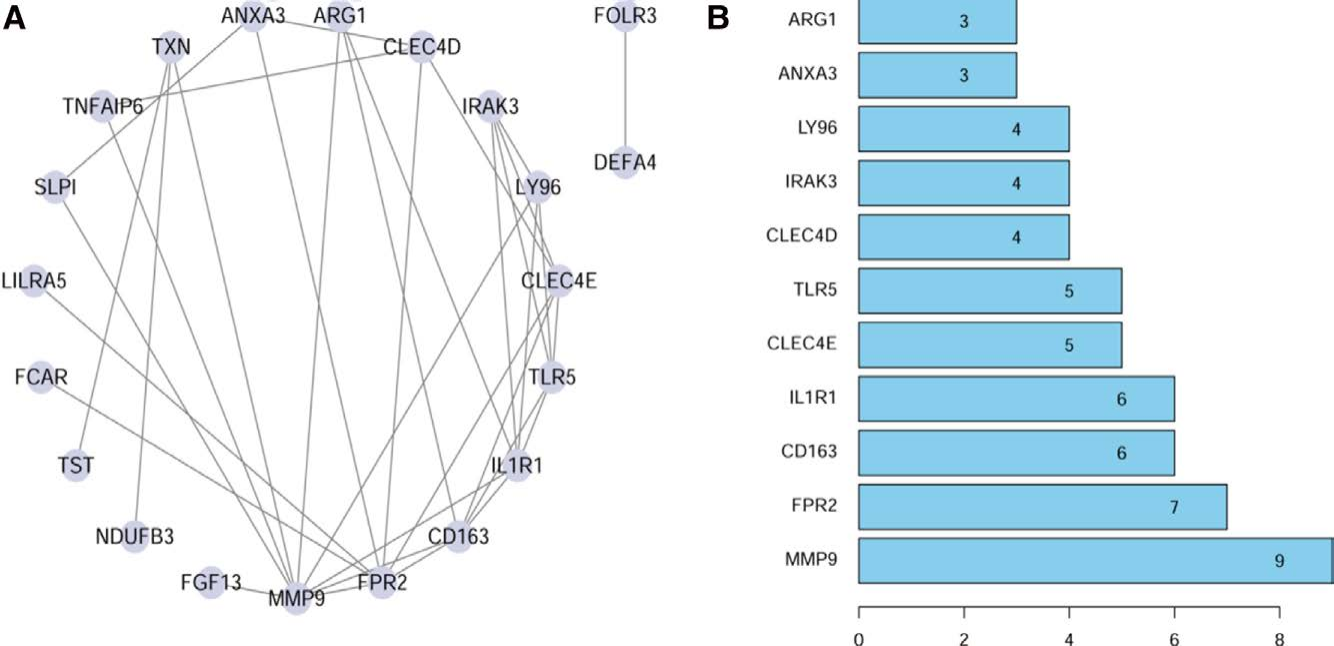
PPI network among DEGs. Panel (A), overlapping DEGs. Panel (B), the number of interactions of overlapping DEGs. ARG1 = arginase-1, CD163 = scavenger receptor cysteine-rich type 1 protein M130, CLEC4D = c-type lectin domain family 4 member D, CLEC4E = c-type lectin domain family 4 member E, DEGs = differentially expressed genes, IL1R1 = interleukin 1 receptor type 1, IRAK3 = interleukin-1 receptor-associated kinase 3, LY96 = lymphocyte antigen 96, MMP9 = matrix metalloproteinase-9, PPI = protein–protein interaction, TLR5 = toll-like receptor 5.

**Figure 9.**
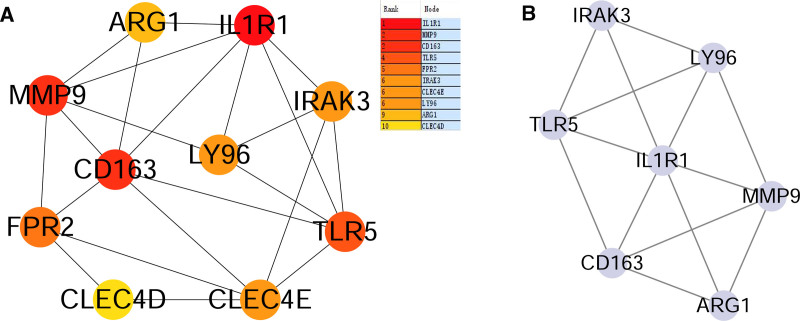
The PPI network analyzed by CytoHubba and MCODE. Panel (A), PPI network for the top 10 hub genes. Panel (B) shows the cluster module extracted using MCODE. PPI = protein–protein interaction.

The PPI network was constructed based on the Interaction Gene Retrieval Tool database and the overlapping DEGs were imported into Cytoscape software (Fig. 8A). The number of interactions was shown in Figure 8B. The Cytoscape plugin CytoHubba was used to analyze the PPI network and identify the hub genes. On the basis of maximum clique centrality algorithm, we identified the top 10 genes as potential hub genes, including interleukin 1 receptor type 1 (IL1R1), matrix metalloproteinase-9 (MMP9), scavenger receptor cysteine-rich type 1 protein M130 (CD163), toll-like receptor 5 (TLR5), N-formyl peptide receptor 2, interleukin-1 receptor-associated kinase 3 (IRAK3), c-type lectin domain family 4 member E (CLEC4E), lymphocyte antigen 96 (LY96), arginase-1 (ARG1), and c-type lectin domain family 4 member D (CLEC4D; Fig. 9A). Subsequently, we identified significant gene cluster modules and obtained clustering scores (filtering criteria: degree cutoff value = 2, node score cutoff value = 0.2, K-core = 2, and max depth = 100) by using the MCODE plugin (Fig. 9B). These modules included 7 hub genes: IRAK3, TLR5, LY96, IL1R1, CD163, MMP9, and ARG1 (Fig. 9B). The hub genes obtained from CytoHubba were intersected with those obtained from MCODE, resulting in 6 hub genes: IL1R1, MMP9, CD163, TLR5, IRAK3, and LY96.

**Figure 10.**
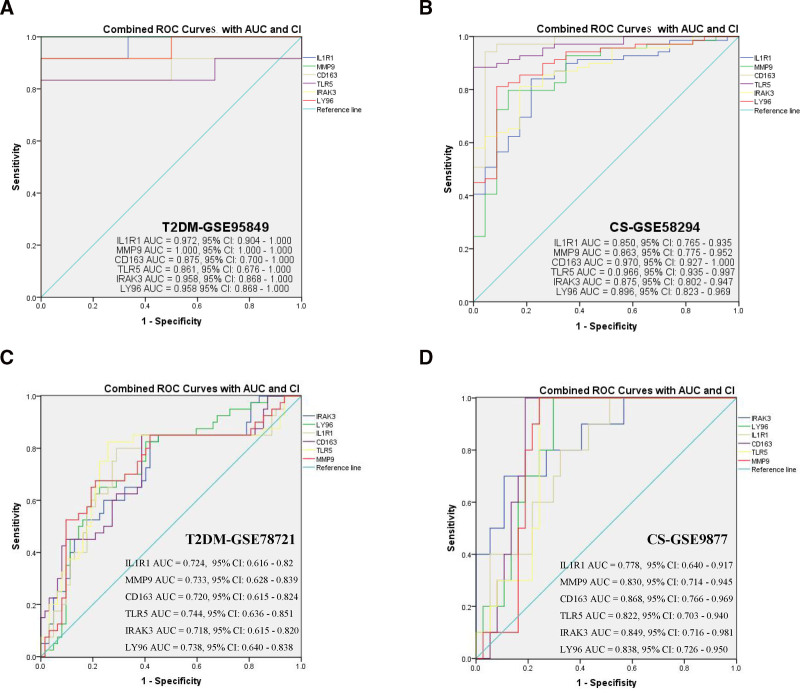
The ROC curves of 6 shared diagnostic markers analyzed in both the T2DM and CS cohorts. (A) ROC curves for 6 shared diagnostic markers in the T2DM-GSE95849 cohort. (B) ROC curves for 6 shared diagnostic markers in the CS-GSE58294 cohort. (C) ROC curves for 6 shared diagnostic markers in the T2DM-GSE78721 cohort. (D) ROC curves for 6 shared diagnostic markers in the CS-GSE9877 cohort. CS = cardiogenic stroke, ROC = receiver operating characteristic, T2DM = type 2 diabetes mellitus.

**Figure 11.**
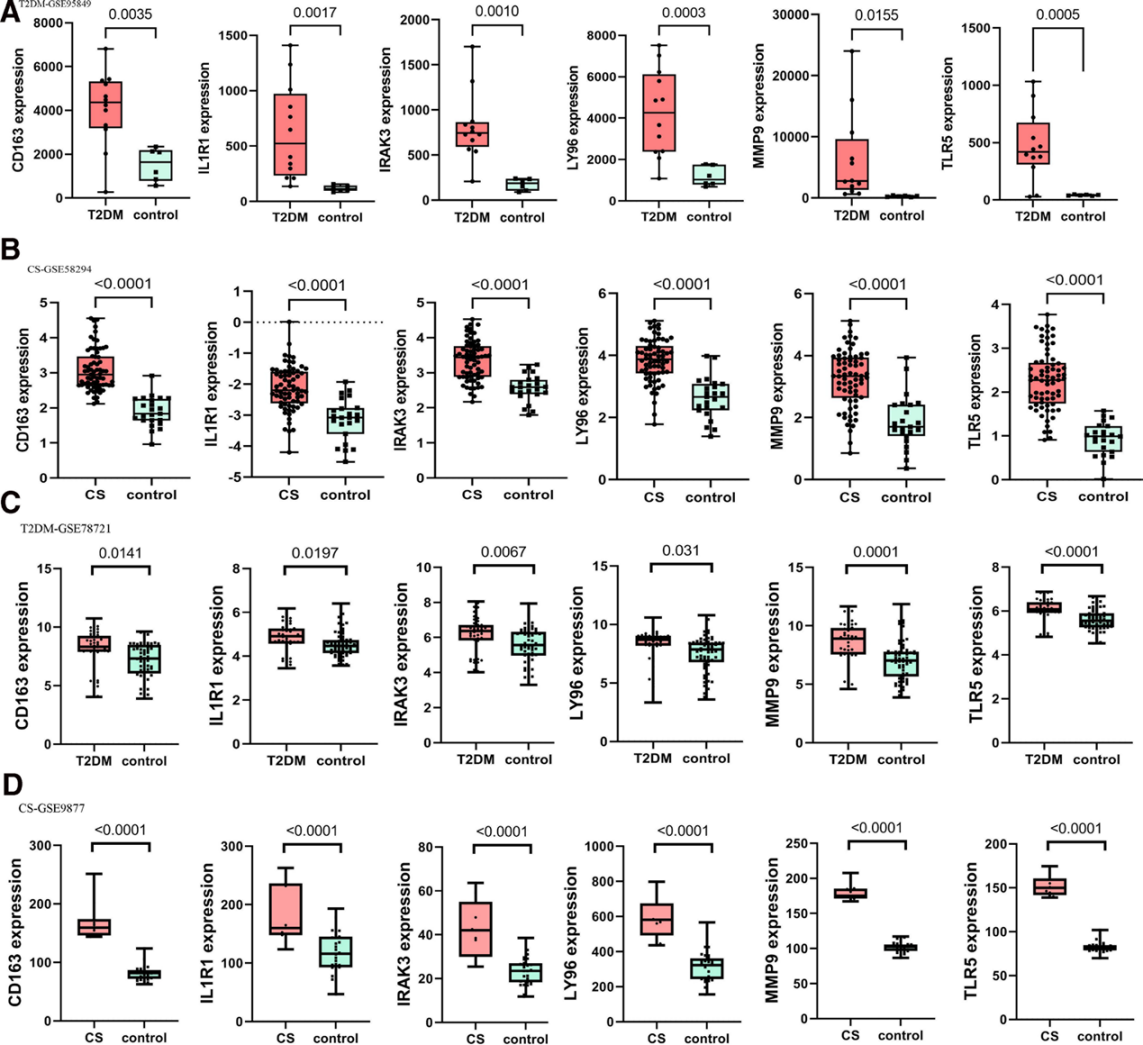
Expression levels of 6 key hub genes analyzed in T2DM (A, C) and CS (B, D). The results show differences in gene expression between normal individuals and those with T2DM/CS. CS = cardiogenic stroke, T2DM = type 2 diabetes mellitus.

In the dataset, the AUC of the different queues also exhibited good predictive performance (Fig. 10). In the T2DM dataset, the AUC for IL1R1, MMP9, CD163, TLR5, IRAK3, and LY96 were 0.972, 1.000, 0.875, 0.861, 0.958, and 0.958, respectively (Fig. 10A). In the CS dataset, all AUCs were >0.8 (Fig. 10B). In the T2DM validation dataset (GSE78721), the AUCs of IL1R1, MMP9, CD163, TLR5, IRAK3, and LY96 were 0.724, 0.733, 0.720, 0.744, 0.718, and 0.738, respectively (Fig. 10C). In the CS validation dataset (GSE9877), the AUCs of all diagnostic biomarkers were >0.7 (Fig. 10D). The box plot shows consistent trends in the differences between the 6 diagnostic indicators in the T2DM and CS datasets (Fig. 11A, B). The validation sets of T2DM and CS also showed consistent differential trends (Fig. 11C, D).

### 3.5. Target miRNA prediction and integrated miRNA target network construction

**Figure 12.**
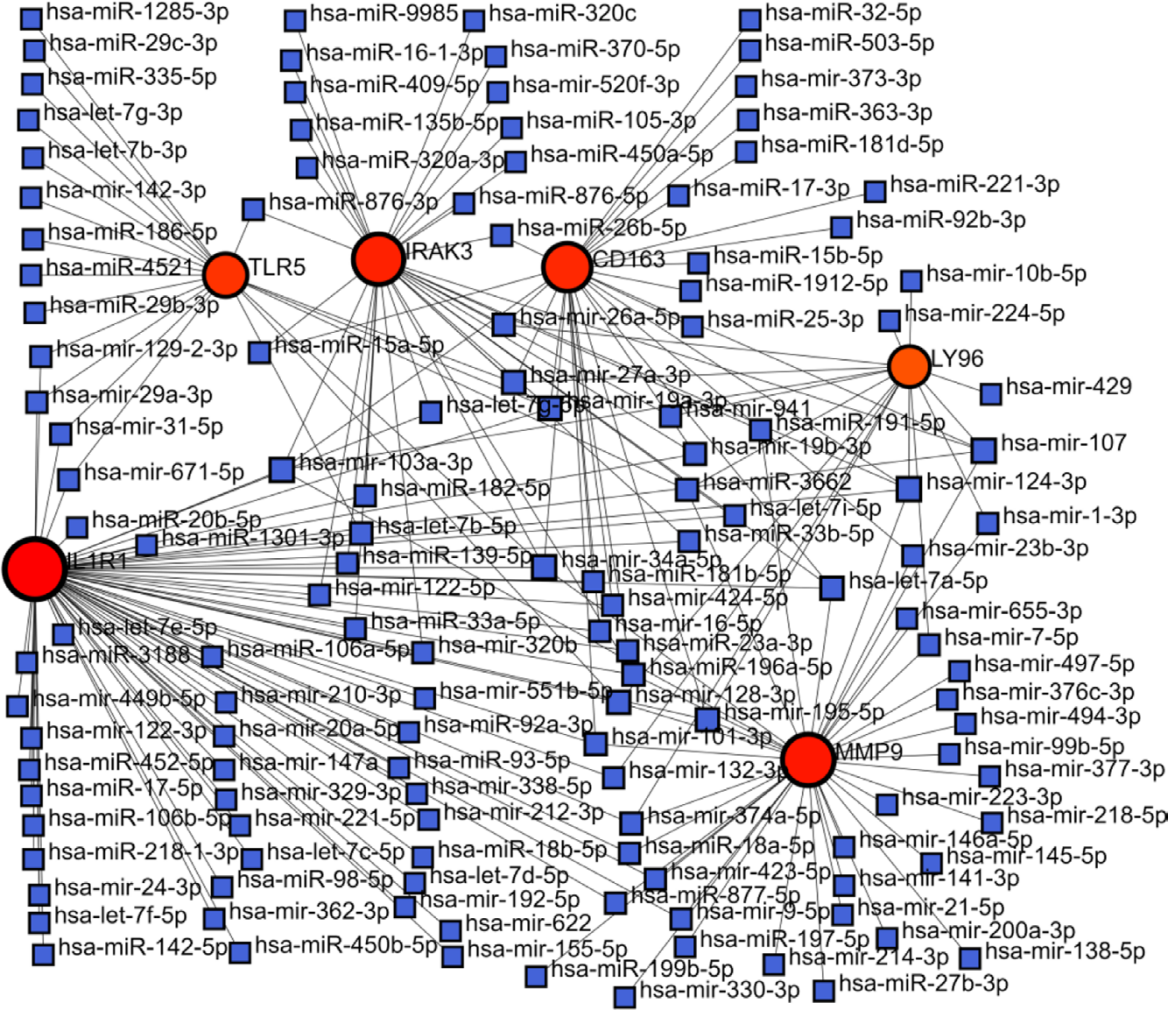
The integrated miRNA-gene interaction networks of 6 hub genes. The red circles symbolize hub genes, while the blue squares represent miRNAs. miRNAs = microRNAs.

We screened the target miRNAs of the hub genes using NetworkAnalyst database. And the miRNA-gene interaction network was constructed using the Cytoscape software. As shown in Figure 12, TLR5 and IL1R1 interacted with miR-671-5p. IRAK3 and CD163 interact with the target miRNA (miR-103a-3p); MiR-124-3p interacts with 2 hub genes, MMP9 and LY96.

### 3.6. Candidate drugs related to hub genes

**Figure 13.**
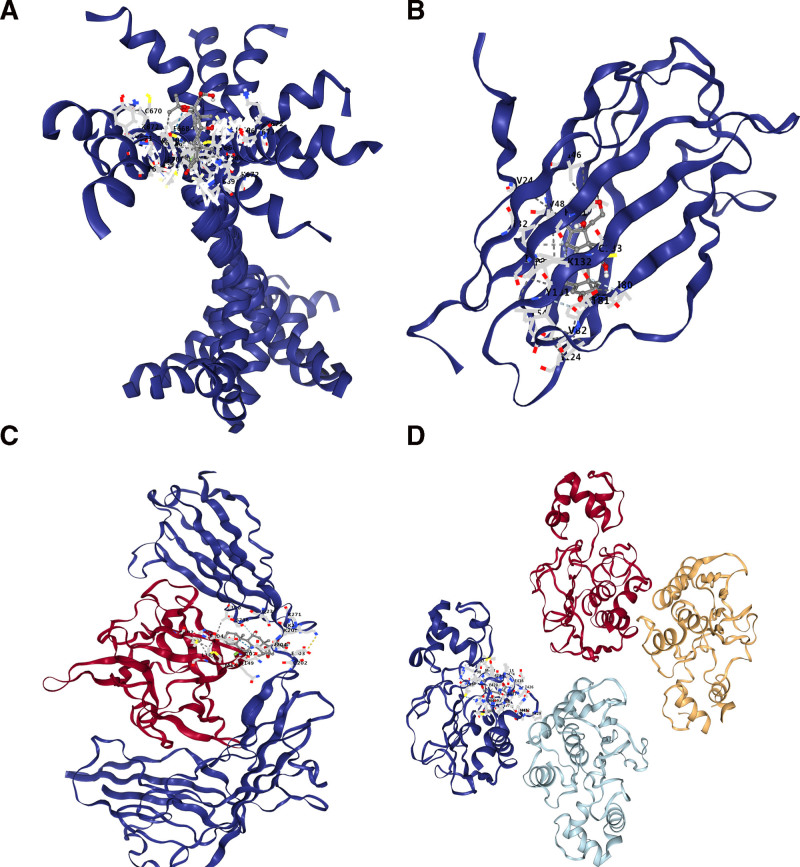
Partial diagram of molecular docking. Panel (A), fludroxycortide-TLR5 molecular docking. Panel (B), flumetasone-LY96 molecular docking. Panel (C), fludroxycortide-IL1R1 molecular docking. Panel (D), molecular docking of d-monapterin and MMP9. IL1R1 = interleukin 1 receptor type 1, LY96 = lymphocyte antigen 96, MMP9 = matrix metalloproteinase-9, TLR5 = toll-like receptor 5.

On the basis of the DSigDB database, 6 candidate drugs (fluoxycortide/flumetasone/d-monapterin/etynodiol/isoflupredone/parthenolide) that were significantly correlated with the hub genes were screened. These candidate drugs might be beneficial for combination therapy of T2DM and CS (Table 2). In addition, we conducted molecular docking on the candidate drugs (Fig. 13). Candidate drugs had good binding with hub genes (Table 3).

**Table 2:** Candidate drugs associated with T2DM and CS.

Drugs	*P*-value	Adjusted *P*-value	Odds ratio
Fludroxycortide HL60 UP	2.83E−05	.003074502	384
Flumetasone HL60 UP	2.83E−05	.003074502	384
d-Monapterin BOSS	3.03E−05	.003074502	369.7592593
Etynodiol HL60 UP	5.92E−07	3.60E−04	332.2333333
Isoflupredone HL60 UP	5.25E−05	.004557618	277.1944444
Parthenolide CTD 00000087	9.50E−05	.007220206	203.5204082

**Table 3:** Binding energies of predicted drugs to the target gene molecules.

Drugs	Binding energies (kcal/mol)					
	MMP9	IL1R1	CD163	TLR5	IRAK3	LY96
Fludroxycortide HL60 UP	−9.1	−9.1	−6.4	−11.5	−8.3	−8.5
Flumetasone HL60 UP	−8.7	−7.4	−6.6	−10.7	−8.6	−8.2
d-Monapterin BOSS	−8.7	−7.2	−5.6	−7.2	−6.6	−5.6
Etynodiol HL60 UP	−8.1	−8.6	−6.0	−11.2	−8.8	−8.9
Isoflupredone HL60 UP	−8.1	−8.1	−6.4	−10.2	−8.3	−8.0
Parthenolide CTD 00000087	−7.4	−7.8	−5.5	−9.3	−8.5	−7.5

CD163 = scavenger receptor cysteine-rich type 1 protein M130, IL1R1 = interleukin 1 receptor type 1, IRAK3 = interleukin-1 receptor-associated kinase 3, LY96 = lymphocyte antigen 96, MMP9 = matrix metalloproteinase-9, TLR5 = toll-like receptor 5.

## 4. Discussion

Several clinical studies have reported the relevance of T2DM and CS. A cohort study^[22]^ reported that in patients with diabetes complicated by atrial fibrillation, a longer diabetic course and higher glycosylated hemoglobin levels increased incidence of stroke. To date, the mechanism underlying the relationship between T2DM and CS isn’t completely clear. It is a matter of great clinical significance to explore the mechanisms of CS in T2DM to facilitate early identification and intervention.

Although the MR-Egger results did not show significant statistical significance, the beta values of the 5 MR methods were consistent in direction, and the IVW and simple model analysis results approve that T2DM enhanced the risk of CS. There was a causal relationship between T2DM and CS, which are consistent with published research findings. To verify the reliability of the MR results, we conducted further testing. The *P* value of Cochran’s *Q* test was >.05, indicating low heterogeneity among SNPs. The *P* values of MR-Egger regression and MR-PRESSO were >.05, indicating that the SNPs closely related to T2DM did not have horizontal pleiotropy.

The results of leave-one-out method further suggested that the MR result was robust. On the basis of GEO database, this study conducted a series of bioinformatics analyses to identify 52 common genes between T2DM and CS. The GO enrichment analysis indicated that GO was mainly enriched in the regulation of monoatomic ion transport, transmembrane transport, defense response to bacteria, and monoatomic ion transmembrane transport. It was indicated that the DEGs between T2DM and CS were bound up with ion transport, immune response, and infection, which was in accord with published researches.^[23]^ KEGG analysis showed that the DEGs were enriched in *S aureus* infection, NF-kappa B signaling pathway, fluid shear stress and atherosclerosis, transcriptional misregulation in cancer, amebiasis, c-type lectin receptor signaling pathway, and sulfur metabolism, mostly related to immunity, inflammation, and infection. People with diabetes are more prone to inflammation and immune responses and are more likely to develop infections.^[24–27]^ The worsening of cardiovascular disease was related to immune inflammatory process and atherosclerosis.^[28]^ *S aureus* increases the incidence rate of infective endocarditis, which is an important cause of CS.^[29–31]^ Therefore, the immune response, inflammatory response, and infection may be key elements in T2DM and CS.

Using Cytoscape’s MCODE and CytoHubba plugins, we selected 6 hub genes in the PPI network: IL1R1, MMP9, CD163, TLR5, IRAK3, and LY96. IL1R1, MMP9, TLR5, and LY96 are upregulated in patients with T2DM, while CD163 and IRAK3 are downregulated. All 6 hub genes were upregulated in patients with CS. These genes may play an important role in the mutual pathogenesis of T2DM and CS.

The proteins encoded by TLR5 recognize bacterial flagellar proteins, which are the main components and virulence factors of bacterial flagella. By activating nuclear factor NF-κB, many inflammation-related target genes were activated. The mutations in TLR5 are associated with susceptibility to legionella. Studies have indicated high blood sugar levels increasing the expressions of TLR1, TLR, TLR2, TLR, TLR4, and TLR5. High blood sugar levels enhance the inflammatory response by increasing the specific features of TLR.^[32]^ Another study showed that TLR5 is associated with vascular dysfunction in obese adults, affecting the visceral fat ratio, fasting insulin, serum IL6, C-reactive protein, and the regulation of arterial blood flow.^[33]^

IRAK3 encodes an interleukin-1 receptor-associated kinase protein that is an important component of the Toll/IL-R immune signaling pathway. IRAK3 is mainly expressed in monocytes and macrophages and acts as a negative regulator of Toll-like receptor signaling. IRAK3 is a key inhibitor of chronic inflammation mediated by NF-κB and is downregulated in monocytes of obese individuals. Low IRAK3 levels are associated with a high incidence of metabolic syndrome, and weight loss is associated with an increase in IRAK3 levels, which is related to a decrease in systemic inflammation and reduction in the number of metabolic syndrome components.^[34]^ Another study showed that, in patients with acute coronary occlusion and ischemic stroke, IRAK3 decreased with increasing NK cell abundance. IRAK3 may have a great impact on NK cell during coronary occlusion and ischemic stroke.^[35]^

CD163 participates in macrophage clearance and endocytosis of hemoglobin/haptoglobin complexes, thereby protecting tissues from oxidative damage mediated by free hemoglobin. The protein encoded by CD163 may also serve as an innate immune sensor for bacteria and induce local inflammation. A systematic evaluation revealed that CD163 is a potential early biomarker of the risk of T2DM. The baseline measurement and longitudinal changes in CD163 positively correlated with insulin resistance; negatively correlated with insulin B cells.^[36]^ CD163 alters complications of diabetes and serum sCD163 levels are a potential biomarker of inflammation. The expression of CD163 on the cell surface and its mRNA in different complications of diabetes are different and require further study.^[37]^ A low circulating CD163 concentration indicates a poor cardiovascular prognosis in patients with and without diabetes.^[38]^

IL1R1 encodes multiple proteins that cause many cytokine induced immune and inflammatory responses. In diabetic patients with hyperglycemia, the methylation status of the CpG site cg13399261 of IL1R1 can be used as an epigenetic marker of chronic inflammation and the development of type 2 diabetes.^[39]^ In acute coronary occlusion and stroke, IL1R1 expression is positively correlated with NK cell abundance. In a mouse trial, IL-1R1 deficiency significantly reduced the infarct size (29%), blood–brain barrier disruption (53%), and neurological deficits (40%). The absence of IL-1 signaling helps improve cerebral blood flow and reduce neutrophil infiltration and vascular activation.^[40]^

LY96 may be involved in endotoxin neutralization. Blocking the TLR4–MD2 complex could reduce blood pressure and improve vascular damage in type 1 diabetes mouse models; however, whether this is effective in type 2 diabetes requires further research.^[41]^ LY96 participating in various biological pathways is interrelated to the activation and regulation of innate immune responses. There is a potential association between LY96 and atrial fibrillation, achieved by affecting NF-κB.^[42]^

MMP9 is associated with type 2 diabetic retinopathy and macroangiopathy, which increase the risk of complications.^[43,44]^ This may be related to 2 SNPs (rs3918242 and rs3918242) in the MMP9 gene promoter.^[45]^

By constructing the miRNA-gene network, we selected 4 key miRNAs (miR-34a-5p, miR-103-3p, miR-107, and miR-124-3p).

Overexpression of miRNA-34a-5p occurred in patients with acute ischemic stroke, and both large- and small-artery stroke types showed elevated miRNA-34a-5p expression. The in vivo experimental results indicated significantly upregulated expression of miRNA-34a-5p in middle cerebral artery occlusion, presenting positive correlation (*r* = 0.742, *P* < .05).^[46]^ MiRNA-34a-5p has potential regulatory effects on acute ischemic stroke and may act as important targets for stroke treatment and recovery.

MiR-103-3p can promote atherosclerosis, regulates endothelial oxidative damage, inhibits the proliferation and differentiation of neural stem cells, and promotes apoptosis by inhibiting lncWDR59.^[47]^ MiR-103-3p is an important target for heart failure. The overexpression of miR-103-3p can exacerbate cardiac hypertrophy.^[48]^

In lipopolysaccharide-induced myocardial cells, an overexpression of miR-107 promotes myocardial cell proliferation, inhibits cell apoptosis, and increases the proportion of stagnant myocardial cells in the S and G2/M phases by inhibiting phosphatase and tensin homolog (PTEN) activation of the PI3K/AKT pathway. This significantly improves the rat cardiac structure changes, reduces inflammatory response, and alleviates damage to the heart caused by sepsis.^[49]^ However, in terms of metabolism, upregulation of miR-103/107 in the liver can lead to insulin resistance.^[50]^

MiR-124-3p can inhibit high glucose-induced endothelial dysfunction by targeting G3BP2 and activating p38MAPK signaling.^[51]^ The expression of hsa-miR-124-3p in the serum of patients with acute ischemic stroke is downregulated. Increased gene expression of has-miR-124-3p decreases serum pro-inflammatory cytokines.^[52]^

In summary, this study determined the correlation between T2DM and CS through MR and bioinformatics analyses as well as the possible biological mechanisms between the 2 diseases. It has been speculated that the miRNA-gene regulatory network is involved in the pathophysiological processes of T2DM and CS, providing a basis for potential diagnostic and therapeutic targets for T2DM and CS.

This study had some limitations. First, relatively few hub genes were identified, and second, the regulatory mechanisms of the hub genes require further verification. Third, the data lacked clinical parameters, such as glycosylated hemoglobin, diabetes course, and diabetes complications. With a small sample size, more external data validation was needed. Moreover, the data source was too singular, with only European populations and a lack of data on other ethnic groups, resulting in overly consistent data.

## 5. Conclusion

Our study analyzed the correlation between T2DM and CS and identified a miRNA-gene network potentially associated with T2DM and CS. We identified 6 hub genes (IL1R1, CD163, TLR5, LY96, MMP9, and IRAK3) and 4 target miRNAs (miR-34a-5p, miR-103-3p, miR-107, and miR-124-3p) that affect the pathophysiological processes of T2DM and CS through ion transport, immune response, and infection. These results will be conducive to the early diagnosis and treatment of T2DM and CS.

Supplemental digital contents “Supplementary Files 1–7” are available for this article (https://links.lww.com/MD/P830; https://links.lww.com/MD/P831; https://links.lww.com/MD/P832; https://links.lww.com/MD/P833; https://links.lww.com/MD/P834).

## Author contributions

**Conceptualization:** Manjia Wang, Min Liu.

**Data curation:** Manjia Wang, Min Liu.

**Formal analysis:** Manjia Wang, Min Liu.

**Funding acquisition:** Min Liu.

**Methodology:** Zhiyuan Deng.

**Project administration:** Hongli Song.

**Software:** Zhiyuan Deng.

**Validation:** Hongli Song.

**Writing – original draft:** Zhiyuan Deng, Min Liu.

**Writing – review & editing:** Min Liu.

## Supplementary Material


